# Clog and Release, and Reverse Motions of DNA in a Nanopore

**DOI:** 10.3390/polym11010084

**Published:** 2019-01-07

**Authors:** Tomoya Kubota, Kento Lloyd, Naoto Sakashita, Seiya Minato, Kentaro Ishida, Toshiyuki Mitsui

**Affiliations:** Department of Mathematics and Physics, Aoyama-Gakuin University, Sagamihara Campus L617, 5-10-1 Fuchinobe, Chuo, Sagamihara, Kanagawa 252-5258, Japan; t_kubota@phys.aoyama.ac.jp (T.K.); klloyd@phys.aoyama.ac.jp (K.L.); Sakashita@phys.aoyama.ac.jp (N.S.); Minato@phys.aoyama.ac.jp (S.M.); k-ishida@phys.aoyama.ac.jp (K.I.)

**Keywords:** translocation, nanopore, DNA, electro-osmosis

## Abstract

Motions of circular and linear DNA molecules of various lengths near a nanopore of 100 or 200 nm diameter were experimentally observed and investigated by fluorescence microscopy. The movement of DNA molecules through nanopores, known as translocation, is mainly driven by electric fields near and inside the pores. We found significant clogging of nanopores by DNA molecules, particularly by circular DNA and linear T4 DNA (165.65 kbp). Here, the probabilities of DNA clogging events, depending on the DNA length and shape—linear or circular—were determined. Furthermore, two distinct DNA motions were observed: clog and release by linear T4 DNA, and a reverse direction motion at the pore entrance by circular DNA, after which both molecules moved away from the pore. Finite element method-based numerical simulations were performed. The results indicated that DNA molecules with pores 100–200 nm in diameter were strongly influenced by opposing hydrodynamic streaming flow, which was further enhanced by bulky DNA configurations.

## 1. Introduction

Single biomolecule detection has been demonstrated in solid-state nanopores and the geometrical configuration of a single biomolecule can be sensed as the molecule passes through the pore (translocates) by analyzing the ionic current profile generated. To drive a biomolecule to translocate and generate an ionic current, an external voltage difference is applied across the pore membrane by a pair of Ag/AgCl electrodes, immersed in *trans* and *cis* reservoirs [1,2,3,4,5,6]. This voltage difference produces an electrophoretic force acting on charged flexible polyelectrolytes of biomolecules not only inside the pore but also around the nanopore, thereby driving them into and through the pore [7]. The selection of pore materials and their functionalization, integration of metal electrodes, and types of ions used in reservoirs are important for translocation experiments. These factors depend on the type of biomolecule used, probing methods, and ionic or tunneling current measurements [8,9,10,11,12,13,14,15,16]. To probe rather large objects such as viruses [17,18], bacteria [19], or protein complexes [12,16], the pore size must be larger than the object. Such pores with diameters of up to a few hundred nanometers may enhance the hydrodynamic effects because of the larger cross-sectional area of the pore, which induces more flux [20]. One such motion generated inside a pore is the electro-osmotic flow [21]. If the pore surface charge is negative, such as in typical nanopore walls of SiN or SiO_2_ membranes, the cation density increases near the nanopore wall and the cations move through the pore while generating flow of the bulk solution under the influence of an electric field applied to drive biomolecules through the pore [22]. The direction of electroosmotic flow is opposite that of DNA translocation, i.e., from *cis* to *trans* reservoirs, because DNA is negatively charged. Such electroosmosis has been directly observed in micro or nano channels, and leads to decreased DNA translocation speed during electrophoresis [23]. Similarly, the decrease in the DNA translocation speed by such hydrodynamic flows can be expected because of the presence of cations that screen negative charges of DNA [24]. This indicates that the presence of DNA inside a pore affects its own translocation dynamics. Such effects would likely be enhanced if the DNA configuration during translocation is complex, such as in hairpin [25,26] or knot formation [27,28].

We observed and analyzed DNA dynamics near a nanopore and its direct translocation by fluorescence microscopy [7,29,30]. Through direct observation, we estimated the electric potential profile near a SiN nanopore [7]. By applying a gate voltage to an Au film on a SiN nanopore membrane, anomalous DNA motions induced by electroosmotic flow via a pore were observed [29]. By using a field-effect transistor nanopore, we developed a method for slowing the translocation speed of DNA by applying pulse voltages only on the field-effect transistor gate electrode, while both ionic solutions in the *trans* and *cis* reservoirs were kept at V = 0 [30]. While performing these experiments, DNA was frequently observed to clog pores with diameters up to 100 nm, terminating the experiments. The clogged DNA is barely removed by sweeping and reversed the bias voltages [7]. Previously, the polymer clogging phenomenon was predicted in pores with sizes smaller than those of polymer knots (i.e., <100 nm for double-stranded DNA) as the geometry of the knots prevents it from passing through the pore [27,28]. Therefore, we examined the occurrence of DNA clogging on our relatively larger pore diameter of approximately 100 nm or more.

We investigated the origin of DNA clogging of pores and prepared pores with diameters of 100 and 200 nm on 200-nm SiN membranes. At this pore size scale, the differences in nanopore geometry, and possibly varying pore-DNA interactions, are minimized. Various DNA lengths and forms, such as circular or linear DNA molecules, were used to study their effects on the clog probability. We determined the clog probabilities of various DNA molecules and evaluated the concurrence of knot formation. Next, we describe two distinct motions of specific DNA molecules near a nanopore. One motion shows how T4 DNA escapes from a pore after clogging the *cis* side of the reservoir, which is the opposite direction of DNA translocation. In the other motion, circular DNA reversed its direction at the nanopore without entering the pore. To evaluate these motions, we simulated the bulk flow induced not only by electroosmosis through the nanopore walls, but also by the presence of DNA inside a pore using finite element analysis. The possibility of stopping DNA translocation through a pore during electrophoresis by enhancing opposing flows by probable DNA configurations inside the pore is discussed. Because of the presence of sensitive interactions between different types of DNA and their various possible configurations in pores at the 100 nm-scale, such nanopores may be useful in biomolecule filtering applications for sorting polymer lengths and separating DNA configurations, such as circular, linear, and knot forms. 

## 2. Materials and Methods

Nanopores were fabricated in 200-nm-thick freestanding silicon nitride (SiN) membranes (40 × 40 μm) by conventional photolithography. The membranes were supported on a 500-μm-thick silicon (100) substrate. A focused ion beam was used to mill 100- or 200 nm-diameter pores and transmission electron microscopy was used to measure the diameter and shape of individual nanopores. The ionic current measurement passing though individual nanopores in the electrolyte solutions was used to confirm the nanopore diameters [9,10]. 

For linear DNA molecules, lambda (48.502 kbp) and T4 (GT7) (166 kbp) were purchased from Nippon Gene Co. (Tokyo, Japan) For 24-kbp DNA, lambda DNA was cut into two 24-kbp (24.5 and 24.0 kbp) fragments by the restriction enzyme XbaI (Takara Bio, Shiga, Japan). To obtain approximately 10-kbp DNA (11.9, 14.3, 9.5, and 12.7 kbp), lambda DNA was digested with the restriction enzyme PVUI (Takara Bio). For circular DNA molecules, phi X174 DNA (5386 bp) were purchased from Nippon Gene Co. To generate 10-kbp circular DNA, 6985-bp DNA fragments were amplified from lambda DNA by PCR using Ex Taq DNA polymerase (Takara Bio) with the specific primers 5′-TTCACTAATGGGCGTGGCTT-3′ and 5′-TTACCAGTTTCCGGCGTACC-3′ (Eurofins Genomics, Louisville, KY, USA). The fragments were purified using the High Pure PCR Cleanup Micro Kit (Roche Applied Science, Basel, Switzerland) and then TA-cloned into the pGEM-T easy vector (3015 bp) (Promega, Madison, WI, USA). The 10-kbp circular DNAs were extracted from *Escherichia coli* and purified using a NucleoBond^®^ Xtra Midi kit (Machery-Nagel, Duren, Germany). The physical properties of these DNAs are summarized in Table S1. YOYO-1, a fluorescent dye, was used to stain the DNA with at a base pair ratio of 1:14, which is lower than the saturation ratio of 1:4, to reduce the mechanical and electrical property changes caused by attaching the dyes [31,32,33]. The final DNA concentration was 1 ng/mL in 0.1 M and 0.01 M KCl solution containing 10 mM Tris-HCl (pH 8.0) and 1 mM EDTA.

The silicon chip containing one or multiple nanopores was placed on a coverslip on the stage of an upright fluorescence microscope (TE2000; Nikon, Tokyo, Japan), which was covered with a PDMS chamber. Our *cis* reservoir was the space between the coverslip and silicon chip where the solution containing fluorescently tagged DNA molecules were injected. A voltage difference of 0.3 V between the *trans* and *cis* reservoirs was applied via Ag/AgCl electrodes to drive DNA molecules from the *trans* to *cis* reservoirs. The stained DNA molecules, illuminated by a 100-W mercury lamp, were detected individually by using two charge-coupled device (CCD) cameras (ORCA-ER and ImageEM X2; Hamamatsu Photonics, Hamamatsu, Japan), which recorded the movement of the shortest DNA molecules, 5 kbp phi X174, in sequential images at 71-ms time intervals. The pixel size differed between these CCD cameras, with values of 0.426 and 0.534 µm for ORCA-ER and ImageEM X2, respectively. Phi X174 DNA was imaged using ImageEM X2, while the other DNA’s were imaged using ORCA-ER in this article. 

The field depth of microscopy at high magnification (i.e., oil immersion objective, 60×, NA 1.4) was less than 1 μm, and thus DNA molecules with a gyration radius of approximately 0.5 μm were sharply imaged within z = ± 0.5 μm of the focusing plane [7]. In this study, the focusing plane for the optical microscope was set to 0.5 μm above the SiN membrane surface on the *cis* side to detect DNA molecules entering a pore. Under this focusing condition, DNA molecules within 1.5 μm of the membrane surface were clearly observed, while the other molecules within 2.0 μm were identified in the plane parallel to the membrane surface. The locations of DNA molecules were identified by evaluating the center geometric means of the pixels of the outlines of DNA. All image processes were performed using MATLAB codes, which were developed in-house. More details regarding our image processing can be found in our previous publications [7,29,30].

To efficiently determine the statistics of clog probabilities, we milled 9 nanopores 20 μm apart in a single freestanding SiN membrane. The separation distance between the pores was longer than twice the capture radius, where DNA motion is affected only by the closest pore, as described below by Equation (1). We used 342 Si chips with more than 2000 nanopores.

Numerical simulations of finite element analysis were performed using a commercial package, COMSOL Multiphysics Software (Version 5.2), with the following three differential equations: Nernst–Planck, Poisson, and Navier–Stokes equations (see details in Figure S1). This is a standard analysis for quantitatively evaluating the bulk flow by electroosmosis and net force acting on DNA molecules [23,34,35,36]. Axisymmetric geometry and solutions were applied to decrease the computation time. For symmetry, we added a linear rod and rings with the surface charge inside a pore simulating a portion of a DNA molecule. 

## 3. Results

### 3.1. Clog Probabilities of Various DNA Length and Configurations 

**Figure 1:**
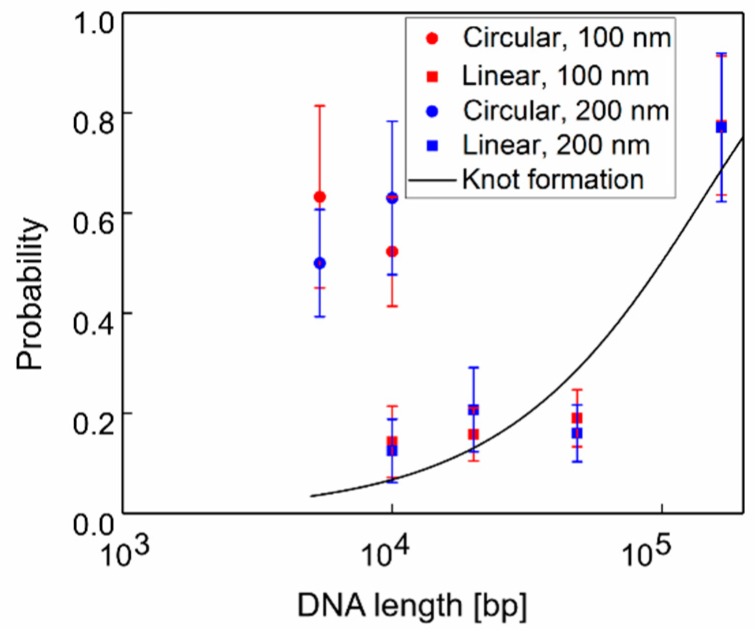
Clog probability vs. DNA length and its shape-linear (squares) or circular (circles) for pore diameters of 100 nm (red) and 200 nm (blue). Error bars represent the standard deviation of each data point. The solid line is the analytical prediction of knot formation, 1−exp(−N/N_0_), where N_0_ = 143 ± 5 kbp by Plesa et al. [27].

As described in our previous publication, translocations through and clogs inside a pore by individual DNA molecules were imaged optically by fluorescence microscopy [7] (see examples in Figure S2). All types of DNA molecules, circular or linear with various lengths, frequently clogged the nanopore. The clogging probability of these DNA molecules are summarized in Figure 1. To evaluate the clogging probability, we defined DNA clog as a DNA molecule in fluorescent images localized at a nanopore for >71 ms in 2 or more sequential image frames at 14 Hz. For example, the expected translocation time of a single lambda DNA translocation was <2 ms for a nanopore with geometry and ionic solutions similar to ours. Thus, DNA located at a pore for >140 ms was considered to bind and clog the pore [9,10,26]. Nearly all linear DNA, except for T4, did not exit the pores toward either the *trans* or *cis* reservoir after binding. When a single DNA molecule clogged a pore, the size of the molecule in the fluorescent images was compressed, likely because a portion of the molecule or the whole molecule entered the pore (see images in Figure S2). The sizes of DNA molecules, estimated from the images, before entering a pore were comparable to those observed previously [37,38].

DNA clogging a pore rarely translocated towards the *trans* reservoirs. Further, most of these molecules were not released from the pore even after reversing the polarity of the applied bias voltage (to push the DNA electrophoretically into the *cis* reservoir). Once a single DNA clogged a pore, the other DNA molecules entering into the pore also caused clogging, as evidenced by the gradual increase in fluorescence intensity as a single DNA entered the pore; this was previously recorded only for lambda DNA [7]. These observations indicate that pore clogging by DNA is a serious concern in nanopore device applications. 

Figure 1 shows that the probability of clogging increases with the length of linear DNA. In contrast, the clogging probabilities for circular DNA appeared to be independent of DNA length. The clogging probability of 10-kbp circular DNA was nearly 3-fold higher than that of linear DNA. Counterintuitively, no significant differences in clogging probabilities were observed between pore diameters, 100 and 200 nm, for either type of DNA molecule. The most interesting comparison is as follows: the quantitative values of the clog probabilities for linear DNA of various lengths were approximately equal to the knot formation probabilities of these linear DNA molecules, which were experimentally estimated by Plesa et al. [27]. This quantitative agreement indicates that clogging occurs because of the presence of DNA knots in a pore. This was unexpected because we used a pore size larger than the typical knot size (size < 100 nm) of 3_1_ to 5_2_ knots, which are mainly formed in DNA [27]. This indicates that the geometrical interaction between a DNA knot and nanopore surface is not the only factor influencing the probability of clogging. There must be a counterforce acting on DNA opposing the electrophoretic force toward the *trans* side, thereby reducing the DNA speed to zero inside a nanopore as the DNA clogs. One possibility is the opposing force of the hydrodynamic flow towards the *cis* side, generated by cations concentrated near the negatively charged DNA molecules inside a pore [24]. The contracting force may be enhanced by DNA knots inside a pore because the multi-fold configuration of a knot may recruit more cations, resulting in the generation of a higher hydrodynamic flow towards the *cis* side. This would also occur for circular DNA, which enters a pore with a two-fold configuration and may show knot occurrence. We determined the motions of DNA under the forces generated by such flows by the finite element method (FEM). Furthermore, we examined the unintuitive DNA motions of T4 DNA and circular DNA, which are likely caused by such counter flows. 

### 3.2. Release of T4 DNA to the cis Side after Clogging

**Figure 2:**
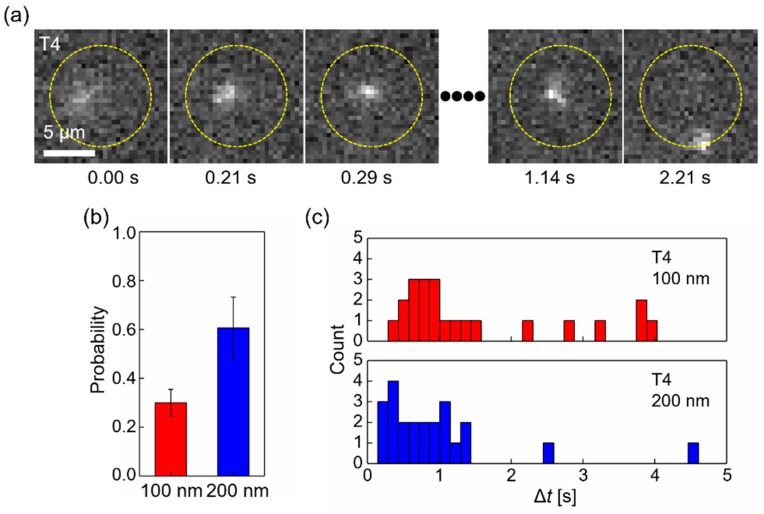
Release of T4 DNA to the *cis* side after clogging a pore. (**a**) Time-resolved fluorescence images of T4 DNA molecules near a nanopore. Scale bar is 5 μm. Images were extracted at *t* = 0.00, 0.21, 0.29, 1.14, and 2.21 s from a sequence of 21 frames recorded at 14 Hz. A DNA approaches a nanopore located at the center in each image marked by a yellow circle. The DNA enters the pore at *t* = 0.29 s, stays clogged at *t* = 1.14 s, and then leaves the pore to the *cis* side. While the DNA clogs the pore, it appears to be stretched out toward the *cis* side at t = 1.14 s. An external bias voltage of 0.3 V was applied to force the DNA to translocate towards the *trans* side during observation (see also Video S1 Releases of T4 DNA). (**b**) Probability of such releasing events after clogging in 100 and 200 nm diameter pores. (**c**) Life-time of clogging for released T4 DNA in 100 and 200 nm diameter pores. The bin size is 0.14 s (2 frames).

As shown in Figure 1a, the clog probability of T4 DNA was nearly 77% for both 100 and 200 nm diameter pores, indicating that the first DNA molecule entering likely becomes clogged. Remarkable motions of T4 DNA, returning single DNA molecules to the *cis* side, were observed after the DNA molecule entered a pore once. Such peculiar motions have not been observed in other linear DNA molecules, such as lambda DNA. Nearly all of these molecules enter the pores and subsequently move within a characteristic distance from the pore, known as the capture radius [25,39,40].

(1) $$r=\frac{\mu E_{pore}r_{pore}^{2}}{4D}$$

where *μ* is electrophoretic mobility, *E_pore_* is the applied electric field strength inside a nanopore, *r_pore_* is the radius of a pore, and *D* is the DNA diffusion constant. The capture radius is estimated by balancing the DNA speed by electrophoresis towards the pore to the average speed by random thermal forces. Briefly, near a pore, a negatively charged DNA molecule is captured by the electric field and then driven towards the pore at a velocity proportional to the electric field with a factor known as electrophoretic mobility in electrophoresis [40]. In contrast, a DNA molecule is constantly moving and changing its direction because of random thermal forces, known as Brownian motion, in a fluid. The capture radius is an analytically estimated distance from a pore, wherein the electrophoresis is a dominant factor [39,40]. For example, the capture radius for lambda DNA is 6.02 μm. Experimentally, within the regions inside of the radius, nearly all lambda DNA molecules, as well as the other DNA types did except for T4 DNA, entered a pore. The capture radius for T4 DNA is 18.7 μm far away from *r* < 7.0 μm where nearly all T4 DNA molecules entered the pore in our observation. This decrease was likely caused by a surface interaction of the large T4 DNA with the membrane [41] because under our focusing conditions, DNA molecules within 2.0 μm from the membrane surface were identified. Removing a T4 DNA from the location of a pore on the *cis* side of reservoir was remarkable. A typical sequence of such a motion is shown in Figure 2a; a T4 DNA moves towards a pore, causing clogging by *t* = 0.29 s, appears to stretch one end of the DNA at *t* = 1.14 s, and then leaves by *t* = 2.21 s (see also Video S1 Release of T4 DNA). Statistically, 30 ± 06% and 61 ± 13% of the clogging DNA was released from the pores toward the *cis* side within 5.0 s (Figure 2b) while the clog probabilities were nearly equal (Figure 1). The strength of the electric fields, 1.08 × 10^6^ V/m (100 nm diameter) and 8.40 × 10^5^ V/m (200 nm diameter) at the pore opening allowed dsDNA in a linear or hairpin formation to pass through the pore as previously reported [27,42,43]. This indicates that a counteracting force to the electrophoretic force acted on the clogged DNA to pull or push it back towards the *cis* side and the force for the 200 nm pore was greater than that of the 100 nm pore. The life-time of clogging, Δ*t*, before release is shown in Figure 2c. Most T4 DNA was released within 1.5 s from either 100 or 200 nm diameter pores. More DNA molecules were released within 0.284 s from the 200 nm diameter pores. Interestingly, DNA was infrequently recaptured and re-entered the pore after its exit. As an example, an SI movie shows T4 DNA being released in a rare recapture event (Video S2 Recapture of released T4 DNA). As shown in these movies, the released DNA remained outside of the region and inside the capture radius. This prevents DNA from recapturing and indicates that the counteracting force can drive released DNA molecules to move out of the capture region. 

### 3.3. U Turn of Circular DNA at Nanopore

**Figure 3:**
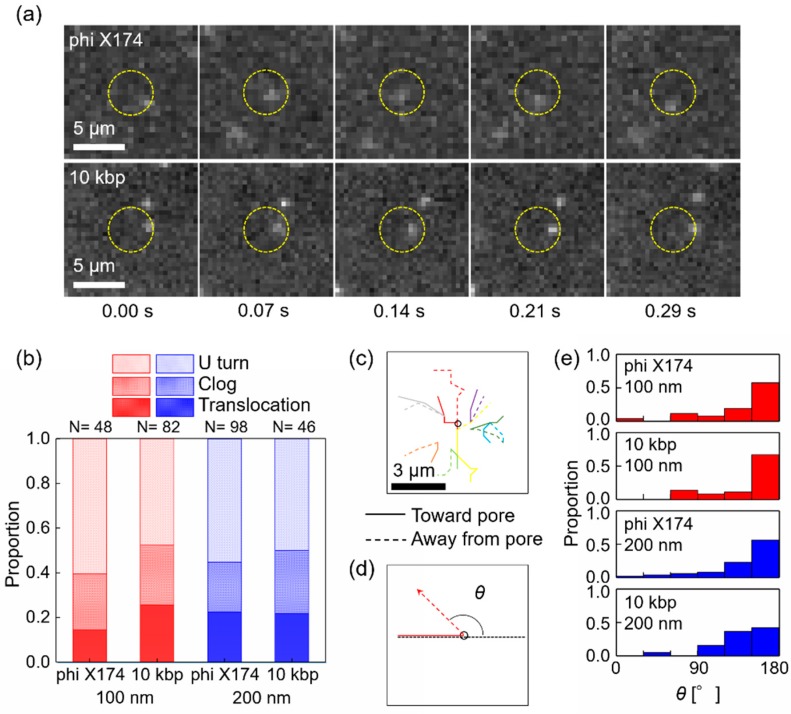
U turn of circular DNA at 100 nm diameter nanopore. (**a**) Two sequences of fluorescence images of a DNA molecule of 10 kbp and phi X174 near nanopore recorded at 14 Hz. Scale bar is 5 μm. A circular DNA molecule moves toward a nanopore within the region inside the capture radius (yellow circle) by *t* = 0.07 s, approaches further at *t* = 0.14 s, and then moves away from the pore after *t* = 0.21 s. An external bias voltage was 0.3 V (see also Video S3, S4 U turn of circular DNA). The images for 10 kbp and phi X174 were acquired with different CCD cameras, altering the pixel counts/scale bar of 5μm (see Materials and Methods). (**b**) Proportion of translocation, clog, and U turn of circular DNA, phi X174 and 10 kbp for 100 and 200 nm diameter pores. Nearly half of DNA U turns. (**c**) Representative trajectories of U turn DNA of 10 kbp. Dotted line shows trajectories moving away from a pore. These DNA molecules mostly turned around at the nanopore. (**d**) A schematic drawing of an angle of deflection at a pore. (**e**) Distributions of the angles of circular DNA for 100 and 200 nm diameter pores.

The other remarkable observations were similar to the clog and release events for T4 DNA. A significant number of circular DNA molecules altered their directions noticeably at nearly the top of a nanopore within regions inside the capture radius and then moved away from the pore without entering it. A typical example of this motion is shown in Figure 3a. In the images, a 10-kbp circular DNA molecule approached a nanopore where *r* < 2.24 μm, which is the capture radius for DNA (yellow circle) by *t* = 0.14 s. The DNA molecule moves to the top of the pore at *t* = 0.21 s. Next, the DNA moved away from the pore. An external bias voltage of 0.3 V was maintained during imaging. The DNA motion in the image sequence appeared to be a “U turn” (see also Video S3, S4 U turn of circular DNA). As shown in Figure 3b, the proportions of this U turn were nearly half of those of the circular DNA, phi X174, and 10 kbp, entering the capture radius from a 100 or 200 nm diameter pore. Representative trajectories of DNA moving away from a 100-nm diameter pore (dotted lines) plotted in Figure 3c showed the U turn motions, altering the direction near 180° towards the pore (solid lines). We defined the angle of the DNA motions between their approach and exit as the pore location at the center as shown schematically in Figure 3d. The distributions of the angles of circular DNA for 100 and 200 nm diameter pores are plotted in Figure 3e. These plots clearly display how the motions eventually formed a U-turn as the peaks changed from 150° to 180°. The average speeds of DNA exit, 15.7 μm/s, from a pore were faster than those of DNA approaching, 8.4 μm/s. Similarly, when T4 DNA was released after clogging as described in the previous section, the instantaneous recapture of leaving DNA was not frequently observed, indicating that the exiting DNA molecules moved out of the capture region once. Again, this U-turn motion indicates that the counteracting force acting on the DNA molecule at a pore was greater than the electrophoretic force driving the DNA into a pore. For circular molecules, the DNA instantaneously moved away from a pore without clogging. Therefore, the direction of circular DNA was altered on or at the opening of a pore rather than inside of a pore. Interestingly, the circular DNA molecules were not released after they clogged a pore, unlike T4 DNA. Instead, both moving away from a pore on the *cis* side should contribute to the counteracting force acting in the opposite direction to the electrophoretic force. The hydrodynamic drag force by electroosmosis can be such a force if the surface charge of a nanopore wall is negative, such as those of SiO_2_ and SiN [22]. However, quantitatively, the force of electroosmosis was smaller than the force of electrophoresis. Because the DNA molecules displayed these motions for T4 (164 kbp) and circular DNA, we expected more multi-folded configurations of negatively charged DNA and a hydrodynamic drag force generated by the presence of DNA in such multi-folded configurations. These configurations likely increased the hydrodynamic flow as observed for multiple DNA molecules inside a pore [24]. 

### 3.4. FEM-Based Numerical Estimation of DNA Motions Near Nanopore 

**Figure 4:**
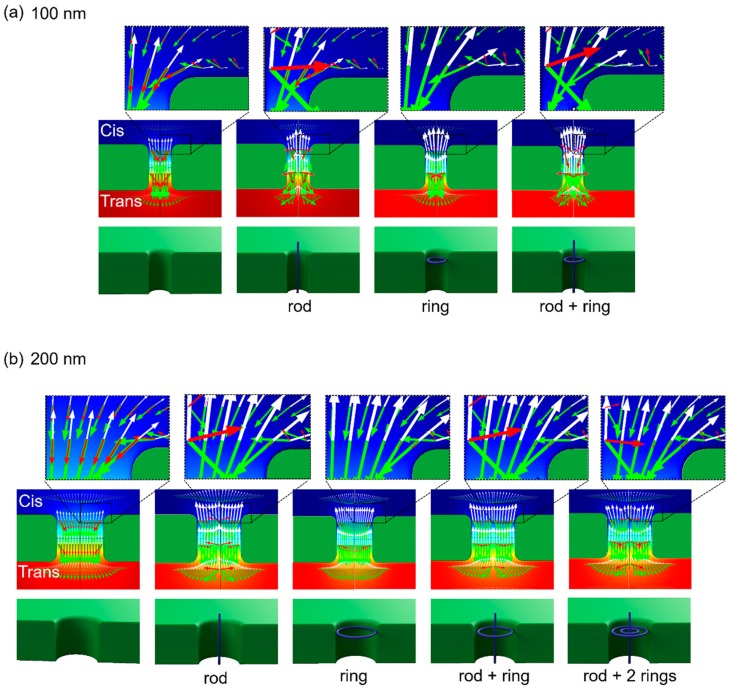
Numerical simulations of DNA motions with the presence of DNA at a nanopore with its diameter, 100 nm (**a**) and 200 nm (**b**). Upper: Zoomed images show the estimated DNA velocities (red arrows) at pore entrance. White arrows represent the velocities of bulk fluidic flow, while green arrows display the velocities by electrophoresis. Middle: Estimated velocities inside a pore. Lower: Schematics indicate the locations of a rod and/or a ring resembling to DNA inside a pore. In the left images simulating no DNA, the red arrows point down towards the *trans* side of expected translocation. In the other images hosting rod and/or ring, the |*v*_z_| of the red arrows decreased not only inside the pore but also at the pore entrances as the oppositely flow velocities (white arrows) increased.

A Finite Element Package, COMSOL Multiphysics Software (Version 5.2), was used to numerically simulate the motion of DNA near and inside a nanopore, particularly the effect of the presence of a multi-folded DNA molecule inside the pore. We previously estimated DNA motions near a nanopore in the presence of electrophoresis and electroosmosis forces [7,29,30]. Briefly, Nernst–Planck, Poisson, and Navier–Stokes equations were used (see Figure S1). Cylindrical symmetry is appropriate for determining the nanopore simulation geometry, resulting in simulation domains defined with axial symmetry in 2 dimensions. The DNA motions were estimated by adding the velocities of electrophoresis and advection [44]. To investigate the effect of the DNA presence around a pore, we located a ring or/and rod, 2 nm in diameter, with schematics depicting the locations in the lower images of Figure 4a,b. The surface charge density of these rings and rods was set to −0.4 e/nm^2^; this value has been used to simulate DNA motion through smaller pores (*r* < 20 nm) in previous studies [45,46]. The estimated DNA velocities around 100 and 200 nm pores with various DNA configurations by FEM are depicted by the red arrows in the middle and upper images of Figure 4. The white arrows represent the velocities of bulk fluidic flow, while the green arrows indicate the velocity by electrophoresis. In the left images of Figure 4a,b, when simulating no DNA inside a pore, the red arrows point down toward the *trans* side, indicating DNA translocation. In contrast, when a hosting rod and/or ring more inside of a pore, flow velocity (white arrows) in the direction opposite of the velocity by electrophoresis (green arrows) increased. As a result, the |*v_z_*| of the net velocities (red arrows) decreased not only inside the pore but also at the pore entrances, as the red arrows are nearly invisible except near a rod in the upper images of Figure 4a,b. Near and, particularly, above the rod, the net velocities (red arrows) point upward, which is opposite the velocities by electrophoresis toward the *trans* side. 

These results indicate that DNA inside of 100 and 200 nm pores which were similar to the rod configurations not only slowed but even reversed the direction of the motion. Such rod and ring configurations inside a pore were likely attained by knots or multi-folded configurations of DNA into our 100 or 200 nm diameter pores, as the size of knots or persistent length of DNA were equal to or below 100 nm. As a result, the DNA molecules with such configurations, initially driving into a pore electrophoretically toward the *trans* side, may stop translocating as clogging or even drive back to the *cis* side mainly because of the hydrodynamic drag force, as we observed.

## 4. Discussion

The results of FEM-based numerical simulation indicated that more DNA rings and rods inside a pore, reflecting the presence of DNA in knot or multi-folded configurations, increases the hydrodynamic bulk flow generated by counterions adjacent to the DNA. This flow likely enhances the drag force that opposes DNA translocation. Larger-diameter pores may produce the same or higher bulk flow depending on the number of DNA files inside the more open entrance of the pore. This prediction based on our numerical simulation results is consistent with our experimental observations. First, the clog probabilities were not influenced by the pore sizes for various DNA molecules. As the probabilities were equivalent to the knot occurrence of linear DNA molecules, the multi-folded configurations of the knot were likely attributable to the clog. Because the number of folded configurations was higher for circular DNA because of its knot formation [47], this DNA was predicted to increase the clog probability, as observed in our results. Furthermore, the two distinct motions of T4 DNA and circular DNA, 10 kbp and phi X174 DNA, respectively, suggest that counter-stream flow towards the *cis* side of the reservoir removes a clogging T4 DNA molecule and effectively ejects a circular DNA molecule, which appeared as a U turn motion in sequential images. First, the longest T4 DNA molecule with the highest probability of forming knots in our experiment assembled at the nanopore entrance, thereby increasing the DNA folds to cause higher counter flow. Such streaming counter flow from a pore may cause stretching of one end of the clogging DNA molecule out of the pore as observed at *t* = 1.14 s (Figure 2a). The higher releasing probability (Figure 2b) and shorter clogging time of T4 DNA before its release (Figure 2c) for the 200 nm diameter pores were also likely related to the higher DNA files or higher counter flow rate from a larger cross-sectional area of the pore, as shown in Figure 4b. Remarkably, T4 DNA showed significant probabilities clogging without exiting a pore. There may be two states of clogging for either linear T4 DNA and circular DNA: one clogs loosely inside the pores, permitting flow in a few seconds, while the other clogs immediately and tightly, similar to linear DNA molecules. However, DNA inside a pore likely encounters hydrodynamic flow. Further studies are necessary to determine the underlying cause of DNA clogs inside a pore and understand how these clogs can be removed. The U turn motion is also required to carry out further experiments and numerical simulations, particularly for FEM in 3D, providing an angle dependence to evaluate the U turn angles. Angles close to 180° were observed in our experiments, indicating that the direction of the counter force on circular DNA by hydrodynamic flow was opposite to the incoming direction of the DNA. The influence of intercalant effects on circular DNA was recently reported in a study using ethidium bromide as a dye molecule [48]. Krueger et al. reported that the formation of branching and supercoiling structures by intercalant ethidium bromide molecules, which increases the number of DNA files when the DNA pass through a pore, were similar to the DNA multi-folded configurations. An atomic force microscopy (AFM) study revealed the supercoiled formation of circular DNA with YOYO-1 dye when the ratio of dye-to-DNA base pair was increased to 1:4 [33]. We cannot exclude such effects; however, our dye-to-DNA base pair ratio was as low as 1:14, which likely reduced the supercoiled formation. Supercoiled DNA was not observed at 1:100 in the AFM study [33]. AFM analysis may be required to quantify the branching or supercoiling formation at 1:14 of the dye-to-DNA base pair ratio to accurately evaluate the portion of U turns of circular DNA in Figure 3b. However, our experimental observations revealed that relatively large pores with diameters from 100 to 200 nm were clogged by DNA molecules. Based on the anomalous dynamics of T4 DNA and circular DNA, our numerical simulations indicate that counter flow generated by adjacent counterions of the DNA through our 100–200 nm diameter pores were susceptive to knot and multi-folded DNA formations or possibly supercoiling caused by dyes, which clog or even exclude these DNAs. Although these large diameter nanopores are not suitable for ionic current measurements to investigate submolecular profiles, the pores can act as filters to remove knots or multi-folded DNA molecules. Such filters may be useful in further screening applications, such as nanopore translocation, for diameters below 5 nm where DNA knots clog the pores immediately [28]. 

## 5. Conclusions

This study was conducted to investigate DNA translocation and pore clogging by circular and linear DNA of various lengths in relatively large nanopores of 100 and 200 nm diameter. DNA molecules were directly observed by fluorescent microscopy. Our results revealed that the probability of clogging increases exponentially with the length of linear DNA and that clogging occurs because of knots or multi-folded DNA configurations entering a pore. Based on our FEM-based numerical simulation, the cause of DNA clogging is hydrodynamic flow, which flows in the direction opposite that of DNA translocation and is mainly generated by cations screening negatively charged DNA inside a pore. The flow acts as a drag force that increases with the number of DNA strands in knot or multi-folded configurations. This concept is consistent with our experimental observations showing that DNA clogging occurs independently of the pore size, with clogging probabilities being higher for circular DNA than for linear DNA. The release of clogged T4 DNA (164 kbp) and the U-turn motion of circular DNA in our observations also agree with this concept. Thus, nanopores with diameters ranging from 100 to 200 nm can be used in applications that filter knots formed by DNA molecules, as they clog these pores.

## Supplementary Materials

The following are available online at http://www.mdpi.com/2073-4360/11/1/84/s1, Figure S1: FEM based numerical simulation, Figure S1: FEM based numerical simulation, Figure S2: Translocation and Clog of DNA, Video S1: Releases of T4 DNA, Video S1: Releases of T4 DNA, Video S2: Recapture of released T4 DNA, Video S3, S4 U turn of circular DNA of 10 kbp (S3) and phi X174 (S4), Video S5 lambda DNA translocate and Video S6 lambda DNA clog. Table S1: Physical properties of DNA.

## References

[1] Dekker C. (2007). Solid-state nanopores. Nat. Nanotechnol..

[2] Branton D., Deamer D.W., Marziali A., Bayley H., Benner S.A., Butler T., Di Ventra M., Garaj S., Hibbs A., Huang X. (2008). The potential and challenges of nanopore sequencing. Nat. Biotechnol..

[3] Deamer D., Akeson M., Branton D. (2016). Three decades of nanopore sequencing. Nat. Biotechnol..

[4] Wanunu M. (2012). Nanopores: A journey towards DNA sequencing. Phys. Life Rev..

[5] Keyser U.F. (2011). Controlling molecular transport through nanopores. J. R. Soc. Interface R. Soc..

[6] Lee K., Park K.B., Kim H.J., Yu J.S., Chae H., Kim H.M., Kim K.B. (2018). Recent Progress in Solid-State Nanopores. Adv. Mater.

[7] Ando G., Hyun C., Li J., Mitsui T. (2012). Directly observing the motion of DNA molecules near solid-state nanopores. ACS Nano.

[8] Fologea D., Ledden B., McNabb D.S., Li J. (2007). Electrical characterization of protein molecules by a solid-state nanopore. Appl. Phys. Lett..

[9] Li J., Gershow M., Stein D., Brandin E., Golovchenko J.A. (2003). DNA molecules and configurations in a solid-state nanopore microscope. Nat. Mater..

[10] Storm A.J., Storm C., Chen J.H., Zandbergen H., Joanny J.F., Dekker C. (2005). Fast DNA translocation through a solid-state nanopore. Nano Lett..

[11] Larkin J., Henley R.Y., Muthukumar M., Rosenstein J.K., Wanunu M. (2014). High-bandwidth protein analysis using solid-state nanopores. Biophys. J..

[12] Talaga D.S., Li J. (2009). Single-molecule protein unfolding in solid state nanopores. J. Am. Chem. Soc..

[13] Tsutsui M., Rahong S., Iizumi Y., Okazaki T., Taniguchi M., Kawai T. (2011). Single-molecule sensing electrode embedded in-plane nanopore. Sci. Rep..

[14] Garaj S., Hubbard W., Reina A., Kong J., Branton D., Golovchenko J.A. (2010). Graphene as a subnanometre trans-electrode membrane. Nature.

[15] Yusko E.C., Johnson J.M., Majd S., Prangkio P., Rollings R.C., Li J., Yang J., Mayer M. (2011). Controlling protein translocation through nanopores with bio-inspired fluid walls. Nat. Nanotechnol..

[16] Han A.P., Schurmann G., Mondin G., Bitterli R.A., Hegelbach N.G., de Rooij N.F., Staufer U. (2006). Sensing protein molecules using nanofabricated pores. Appl. Phys. Lett..

[17] McMullen A., de Haan H.W., Tang J.X., Stein D. (2014). Stiff filamentous virus translocations through solid-state nanopores. Nat. Commun..

[18] Arima A., Tsutsui M., Harlisa I.H., Yoshida T., Tanaka M., Yokota K., Tonomura W., Taniguchi M., Okochi M., Washio T. (2018). Selective detections of single-viruses using solid-state nanopores. Sci. Rep..

[19] Ryuzaki S., Tsutsui M., He Y., Yokota K., Arima A., Morikawa T., Taniguchi M., Kawai T. (2017). Rapid structural analysis of nanomaterials in aqueous solutions. Nanotechnology.

[20] Bell N.A.W., Chen K., Ghosal S., Ricci M., Keyser U.F. (2017). Asymmetric dynamics of DNA entering and exiting a strongly confining nanopore. Nat. Commun..

[21] Squires T.M., Quake S.R. (2005). Microfluidics: Fluid physics at the nanoliter scale. Rev. Mod. Phys..

[22] Wong C.T., Muthukumar M. (2007). Polymer capture by electro-osmotic flow of oppositely charged nanopores. J. Chem. Phys..

[23] van der Heyden F.H., Bonthuis D.J., Stein D., Meyer C., Dekker C. (2006). Electrokinetic energy conversion efficiency in nanofluidic channels. Nano Lett..

[24] Laohakunakorn N., Ghosal S., Otto O., Misiunas K., Keyser U.F. (2013). DNA interactions in crowded nanopores. Nano Lett..

[25] Chen P., Gu J., Brandin E., Kim Y.R., Wang Q., Branton D. (2004). Probing Single DNA Molecule Transport Using Fabricated Nanopores. Nano Lett..

[26] Mihovilovic M., Hagerty N., Stein D. (2013). Statistics of DNA capture by a solid-state nanopore. Phys. Rev. Lett..

[27] Plesa C., Verschueren D., Pud S., van der Torre J., Ruitenberg J.W., Witteveen M.J., Jonsson M.P., Grosberg A.Y., Rabin Y., Dekker C. (2016). Direct observation of DNA knots using a solid-state nanopore. Nat. Nanotechnol..

[28] Rosa A., Di Ventra M., Micheletti C. (2012). Topological jamming of spontaneously knotted polyelectrolyte chains driven through a nanopore. Phys. Rev. Lett..

[29] Sugimoto M., Kato Y., Ishida K., Hyun C., Li J., Mitsui T. (2015). DNA motion induced by electrokinetic flow near an Au coated nanopore surface as voltage controlled gate. Nanotechnology.

[30] Kato Y., Sakashita N., Ishida K., Mitsui T. (2018). Gate-Voltage-Controlled Threading DNA into Transistor Nanopores. J. Phys. Chem. B.

[31] Gunther K., Mertig M., Seidel R. (2010). Mechanical and structural properties of YOYO-1 complexed DNA. Nucleic Acids Res..

[32] Zhang C., Zhang F., van Kan J.A., van der Maarel J.R. (2008). Effects of electrostatic screening on the conformation of single DNA molecules confined in a nanochannel. J. Chem. Phys..

[33] Kundukad B., Yan J., Doyle P.S. (2014). Effect of YOYO-1 on the mechanical properties of DNA. Soft Matter.

[34] Mao M., Ghosal S., Hu G. (2013). Hydrodynamic flow in the vicinity of a nanopore induced by an applied voltage. Nanotechnology.

[35] Constantin D., Siwy Z.S. (2007). Poisson-Nernst-Planck model of ion current rectification through a nanofluidic diode. Phys. Rev. E Stat. Nonline Soft Matter Phys..

[36] He Y., Tsutsui M., Fan C., Taniguchi M., Kawai T. (2011). Controlling DNA translocation through gate modulation of nanopore wall surface charges. ACS Nano.

[37] Hsieh C.C., Balducci A., Doyle P.S. (2008). Ionic effects on the equilibrium dynamics of DNA confined in nanoslits. Nano Lett..

[38] Dorfman K.D. (2010). DNA electrophoresis in microfabricated devices. Rev. Mod. Phys..

[39] Gershow M., Golovchenko J.A. (2007). Recapturing and trapping single molecules with a solid-state nanopore. Nat. Nanotechnol..

[40] Nakane J., Akeson M., Marziali A. (2002). Evaluation of nanopores as candidates for electronic analyte detection. Electrophoresis.

[41] Dai L., Renner C.B., Doyle P.S. (2016). The polymer physics of single DNA confined in nanochannels. Adv. Colloid Interface Sci..

[42] Vlassarev D.M., Golovchenko J.A. (2012). Trapping DNA near a solid-state nanopore. Biophys. J..

[43] Chen L., Conlisk A.T. (2011). Forces affecting double-stranded DNA translocation through synthetic nanopores. Biomed. Microdevices.

[44] Stein D., Deurvorst Z., van der Heyden F.H., Koopmans W.J., Gabel A., Dekker C. (2010). Electrokinetic concentration of DNA polymers in nanofluidic channels. Nano Lett..

[45] Mitscha-Baude G., Buttinger-Kreuzhuber A., Tulzer G., Heitzinger C. (2017). Adaptive and iterative methods for simulations of nanopores with the PNP–Stokes equations. J. Comput. Phys..

[46] Buyukdagli S., Ala-Nissila T. (2014). Controlling Polymer Translocation and Ion Transport via Charge Correlations. Langmuir.

[47] Suma A., Micheletti C. (2017). Pore translocation of knotted DNA rings. Proc. Natl. Acad. Sci. USA.

[48] Krueger E., Shim J., Fathizadeh A., Chang A.N., Subei B., Yocham K.M., Davis P.H., Graugnard E., Khalili-Araghi F., Bashir R. (2016). Modeling and Analysis of Intercalant Effects on Circular DNA Conformation. ACS Nano.

