# Supplementary material for: Clog and Release, and Reverse Motions of DNA in a Nanopore
Source: Polymers (Basel). 2019 Jan 7;11(1):84. doi: 10.3390/polym11010084 (PMC6401990; doi:10.3390/polym11010084)
Supplement: Supplementary file 1 [file polymers-11-00084-s001.zip › polymer_kubota_mitsui_JPN_20180107SI/polymer_kubota_mitsui_aoyama_20181123SI01.pdf]

Supplementary Materials:

Clog and release, and reverse motions of DNA in a nanopore

Tomoya Kubota[1], Kent Lloyd[1], Naoto Sakashita[1], Seiya Minato[1], Kentaro Ishida[1], and Toshiyuki Mitsui[1,*]

Figure S1. FEM based numerical simulation

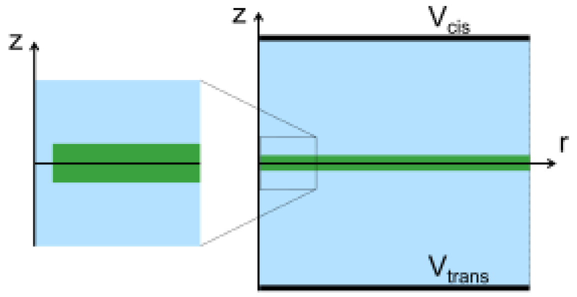

As shown in Figure S1 for our nanopore simulation geometry, the thickness of the freestanding SiN membrane was set as 200 nm and the pore's inner radius was either 20 nm or 100nm for 100 nm and 200 nm diameter pores, respectively. The simulation size was 20 × 20 μm which corresponds to 0 ≤ r ≤ 20 μm with -10 ≤ z ≤ 10μm in the cylindrical coordinate. As for boundary conditions, the locations where electrical potentials for the cis and trans electrodes are also shown as lines, z = 10μm and z = -10 μm, respectively. The electric fields driving DNA molecules electrophoretically around a nanopore and the possible bulk fluid motions by electroosmosis can be estimated by three equations, Nernst-Planck equation (S1), Poisson equation (S2) and Navier-Stokes equation (S3),

$$\frac{\partial c_i}{\partial t} = -\nabla \cdot (\boldsymbol{v} - D_i \nabla c_i - \mu_i c_i \nabla \phi) \qquad \text{S1}$$

$$\nabla^2 \phi = -\frac{\rho_e}{\varepsilon} = -\frac{e \sum z_i c_i}{\varepsilon} \qquad \text{S2}$$

$$\rho \left(\frac{\partial v}{\partial t} + (\boldsymbol{v} \cdot \nabla) \boldsymbol{v} \right) = -\nabla p + \eta \nabla^2 \boldsymbol{v} + \frac{e \sum z_i c_i}{\varepsilon} \nabla \phi \qquad \text{S3}$$

where, c_i, D_i μ_i and z_i are the concentration, diffusion constant, ionic mobility and ionic valence for ion kinds i, respectively. Φ, ρ, $\boldsymbol{v}$ and p are the local electric potential, fluid density, fluid velocity and hydrostatic pressure, respectively. ρ_e, e, ε and η are the charge density, elementary charge, permittivity and liquid viscosity, respectively. The ionic concentration was increased by 5 mM to compensate the ionic strength of TE buffer in solution. The velocity of the DNA motion driven by

electrophoresis can be estimated by $v_{ele} = \mu_{DNA} \nabla\Phi$ where μ_{DNA} is DNA's mobility. The velocity induced by the fluidic motion i.e. electroosmosis is v in Eq. S3. The net DNA velocity can be estimated by adding these velocities.

Figure S2. Translocation and Clog of DNA

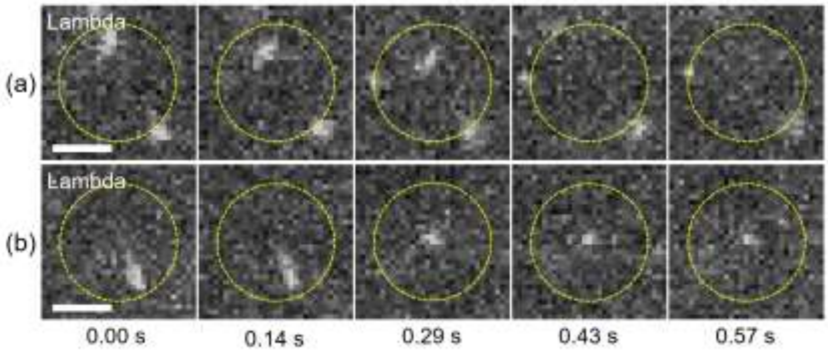

Representative time-resolved fluorescence images of a lambda DNA molecule near a nanopore, displaying (a) translocation and (b) clog. Images are extracted from a sequence of frames recorded at 14 Hz. See also Video S5 lambda DNA translocate and Video S6 lambda DNA clog.

Table S1. Physical properties of DNA

.

DNA	DNA length [kbp]	Diffusion coefficient [μm²/s]	Mobility [10^{-8} m²/Vs]
Phi X174	5.39	1.5 [1, 2]	3.8 [3]
10 kbp circular	10.0	3.8 [2]	
10 kbp	10.0	3.5	1.1 [4]
λ	48.5	0.47 [5]	4.2 [5]
T4	166	0.22 [6]	2.8 [7]

1. Stellwagen, N.C.; Gelfi, C.; Righetti, P.G. The free solution mobility of DNA. *Biopolymers* **1997**, *42*, 687-703, doi:10.1002/(SICI)1097-0282(199711)42:6<687::AID-BIP7>3.0.CO;2-Q [pii] 10.1002/(SICI)1097-0282(199711)42:6<687::AID-BIP7>3.0.CO;2-Q.
2. Lin, P.K.; Chang, J.F.; Wei, C.H.; Tsao, P.H.; Fann, W.S.; Chen, Y.L. Partial hydrodynamic screening of confined linear and circular double-stranded DNA dynamics. *Physical Review E* **2011**, *84*, 031917, doi:10.1103/PhysRevE.84.031917.
3. Robertson, R.M.; Laib, S.; Smith, D.E. Diffusion of isolated DNA molecules: Dependence on length and topology. *Proceedings of the National Academy of Sciences* **2006**, *103*, 7310.

4. Cross, J.D.; Strychalski, E.A.; Craighead, H.G. Size-dependent DNA mobility in nanochannels. *Journal of Applied Physics* **2007**, *102*, 024701, doi:10.1063/1.2757202.
5. Nkodo, A.E.; Garnier, J.M.; Tinland, B.; Ren, H.; Desruisseaux, C.; McCormick, L.C.; Drouin, G.; Slater, G.W. Diffusion coefficient of DNA molecules during free solution electrophoresis. *Electrophoresis* **2001**, *22*, 2424-2432, doi:10.1002/1522-2683(200107)22:12<2424::AID-ELPS2424>3.0.CO;2-1.
6. Balducci, A.; Mao, P.; Han, J.Y.; Doyle, P.S. Double-stranded DNA diffusion in slitlike nanochannels. *Macromolecules* **2006**, *39*, 6273-6281, doi:Doi 10.1021/Ma061047t.
7. Gyurova, A.Y.; Michna, A.; Nikolov, L.; Mileva, E. Self-assembly of four- and two-antennary oligoglycines in aqueous medium. *Colloids and Surfaces A: Physicochemical and Engineering Aspects* **2017**, *519*, 106-116, doi:https://doi.org/10.1016/j.colsurfa.2016.09.012.
