# Supplementary figures and images for: Clog and Release, and Reverse Motions of DNA in a Nanopore
Source: Polymers (Basel). 2019 Jan 7;11(1):84. doi: 10.3390/polym11010084 (PMC6401990; doi:10.3390/polym11010084)

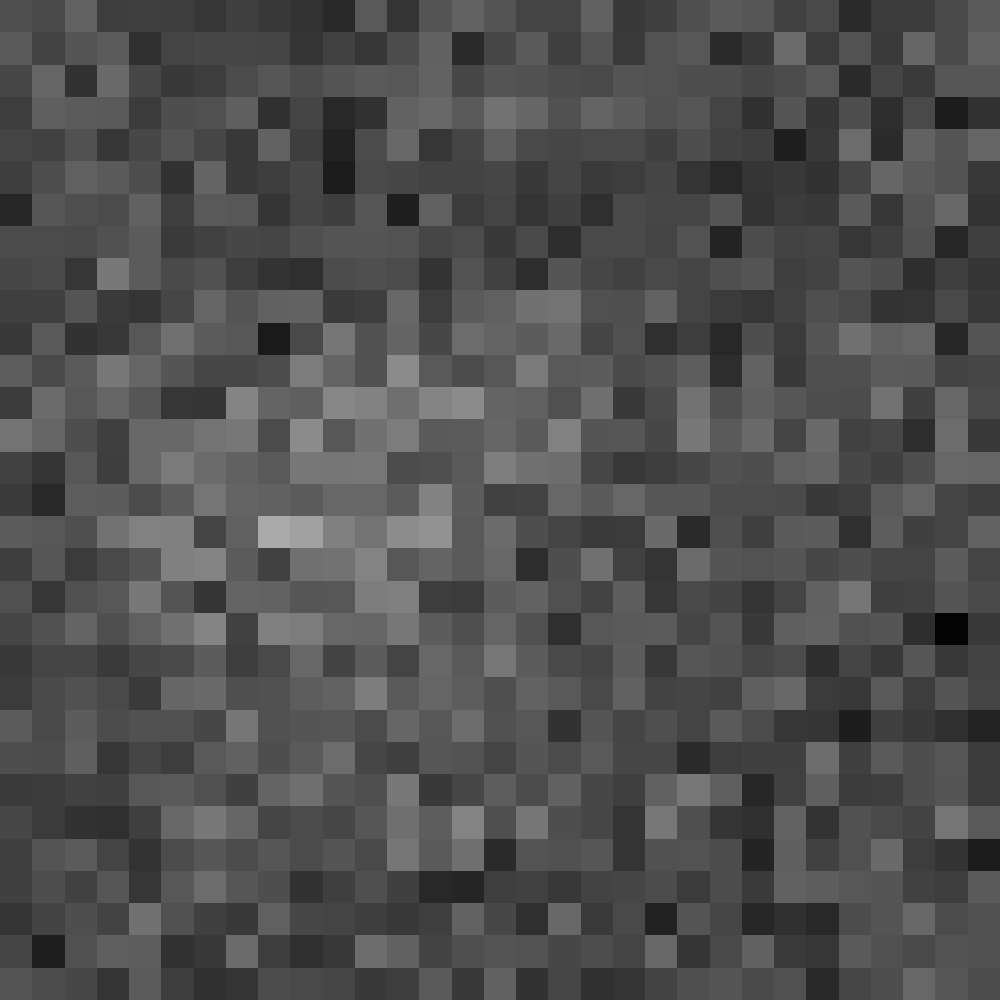

Supplement: Supplementary file 1 [file polymers-11-00084-s001.zip › polymer_kubota_mitsui_JPN_20180107SI/Video S1 Release of T4 DNA.gif]

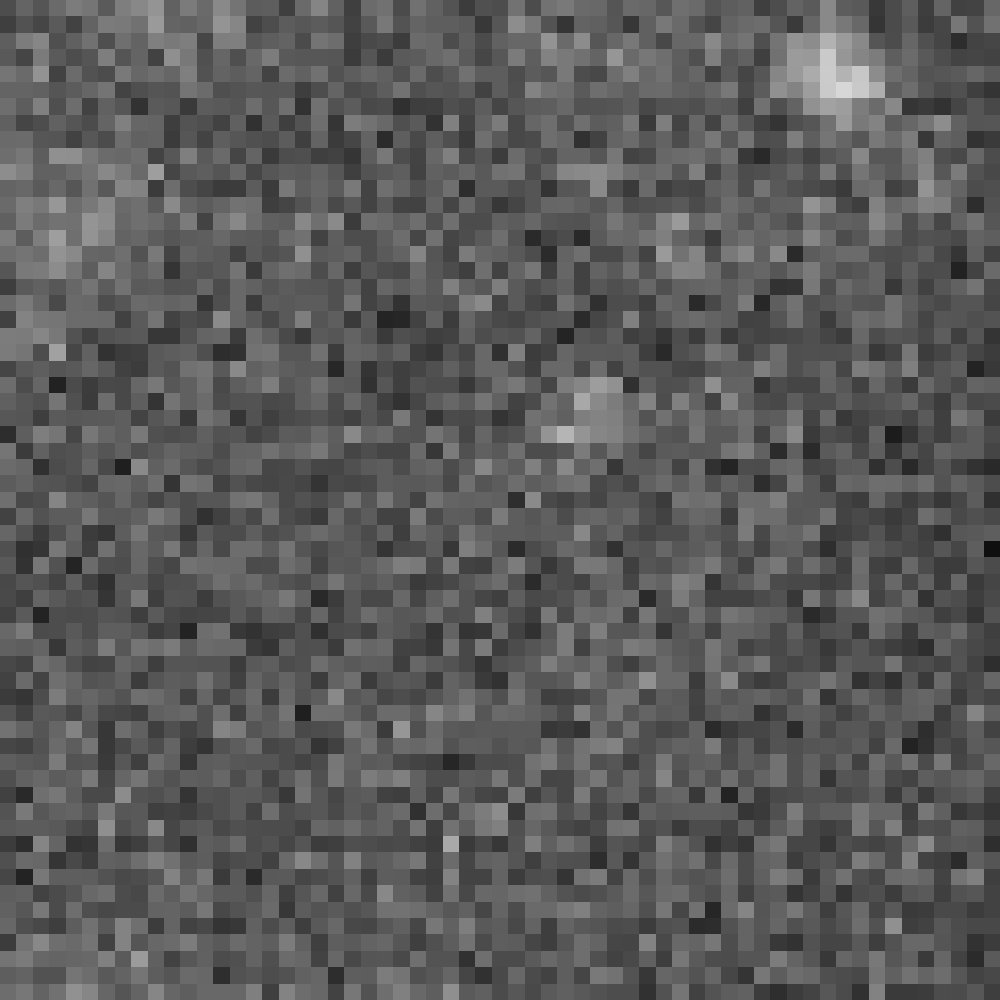

Supplement: Supplementary file 1 [file polymers-11-00084-s001.zip › polymer_kubota_mitsui_JPN_20180107SI/Video S2 Recapture of released T4 DNA.gif]

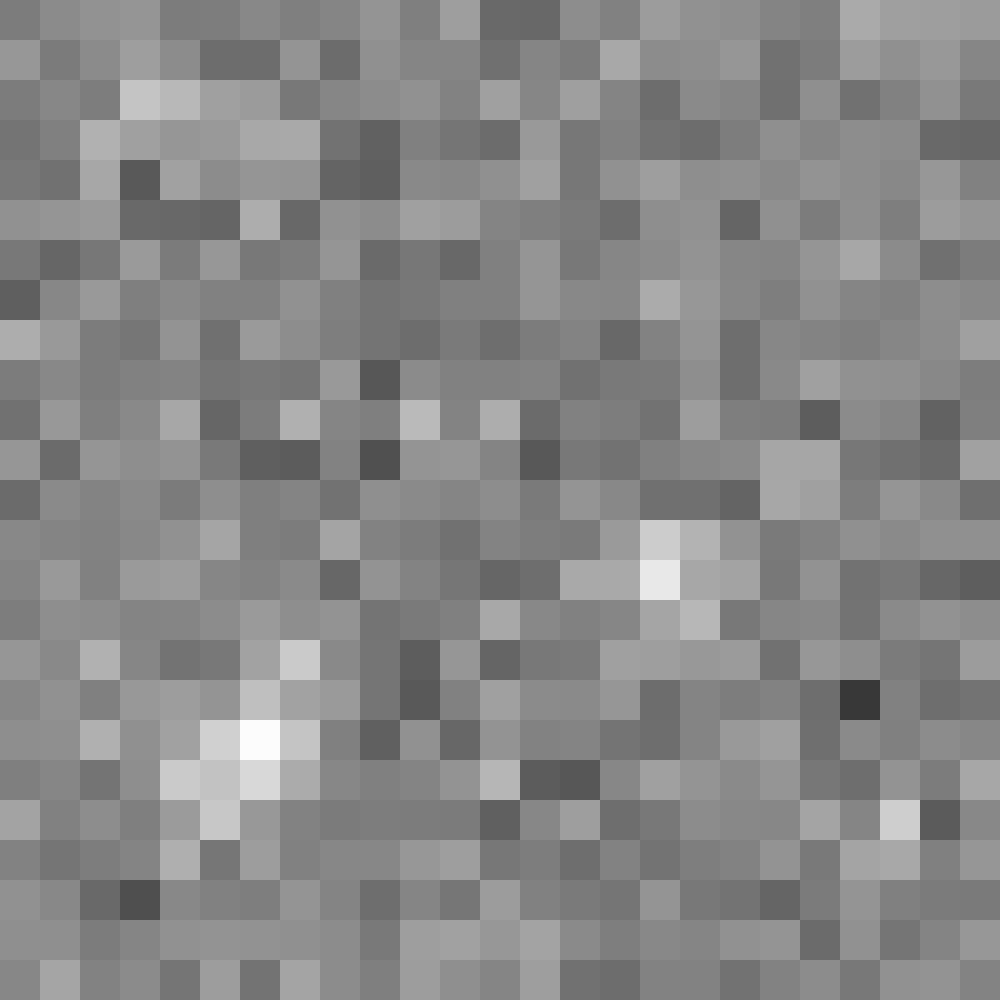

Supplement: Supplementary file 1 [file polymers-11-00084-s001.zip › polymer_kubota_mitsui_JPN_20180107SI/Video S3 U turn of phi X174 DNA.gif]

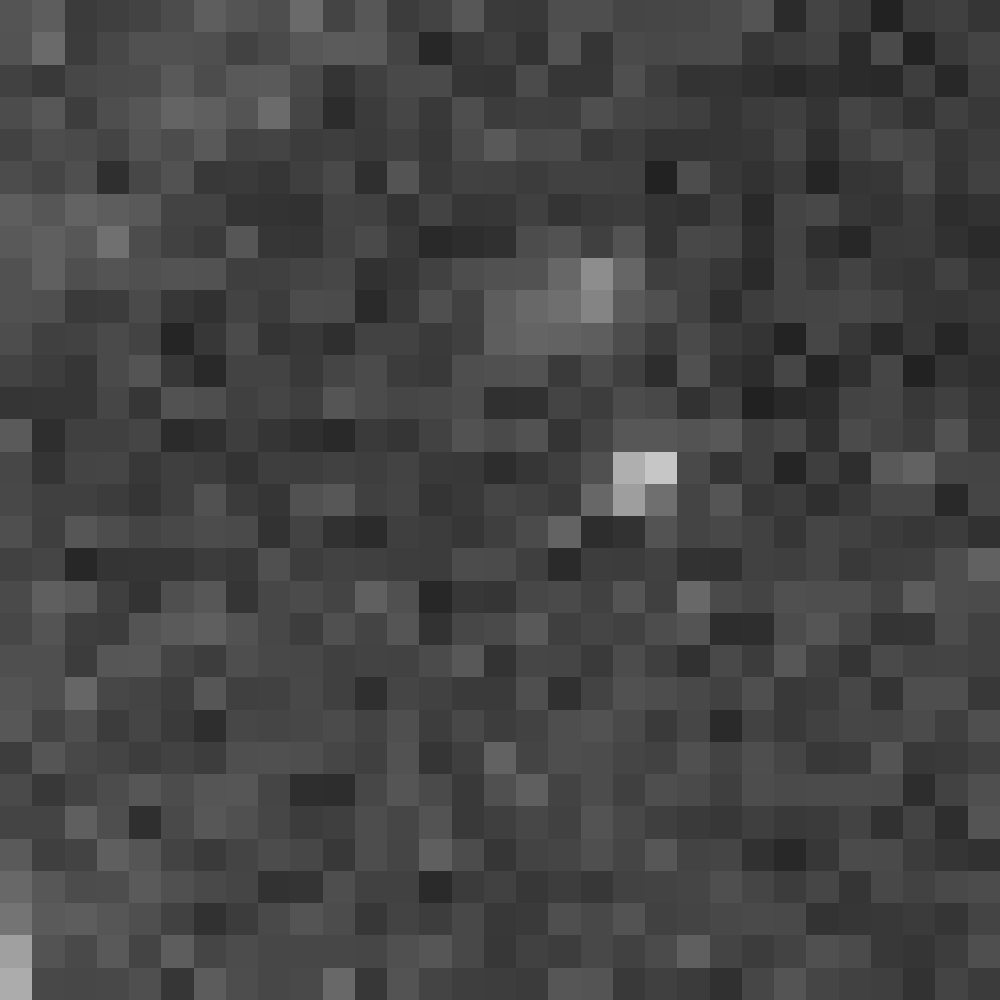

Supplement: Supplementary file 1 [file polymers-11-00084-s001.zip › polymer_kubota_mitsui_JPN_20180107SI/Video S4 U turn of 10kbp DNA.gif]

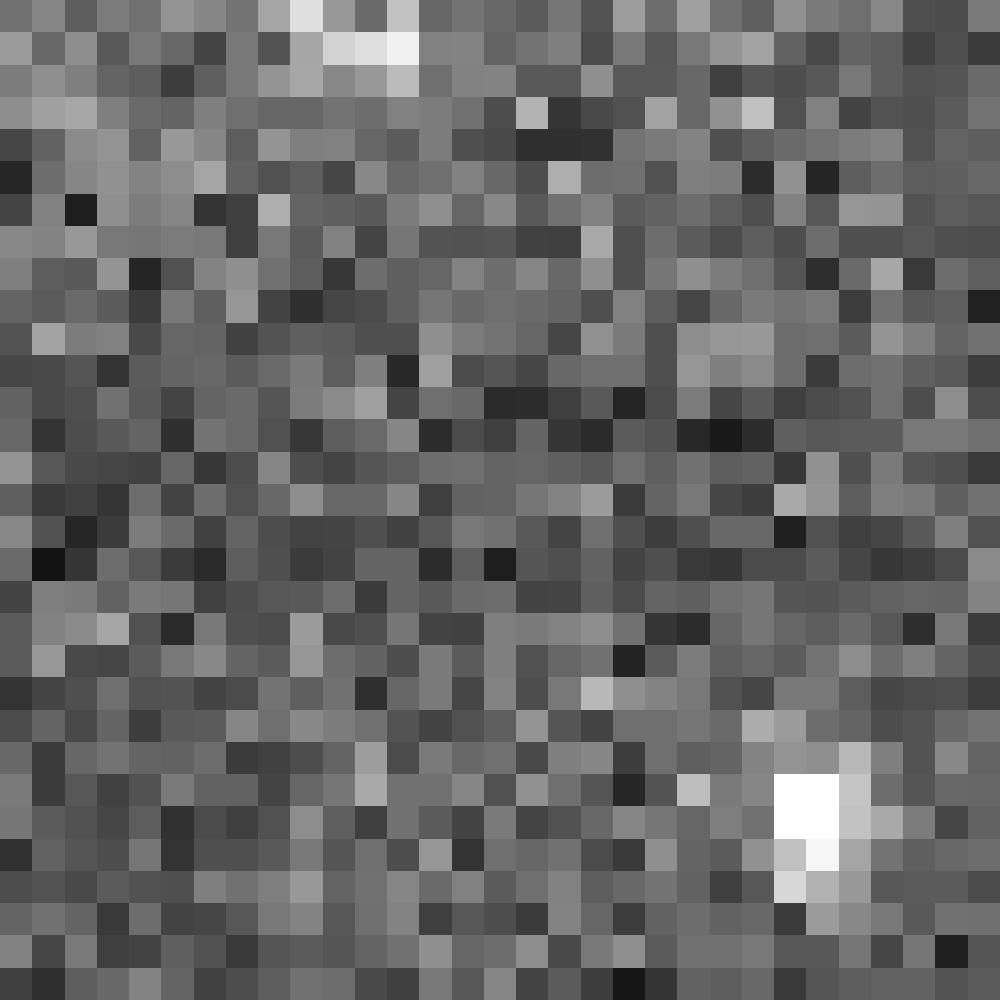

Supplement: Supplementary file 1 [file polymers-11-00084-s001.zip › polymer_kubota_mitsui_JPN_20180107SI/Video S5 lambda DNA translocate.gif]

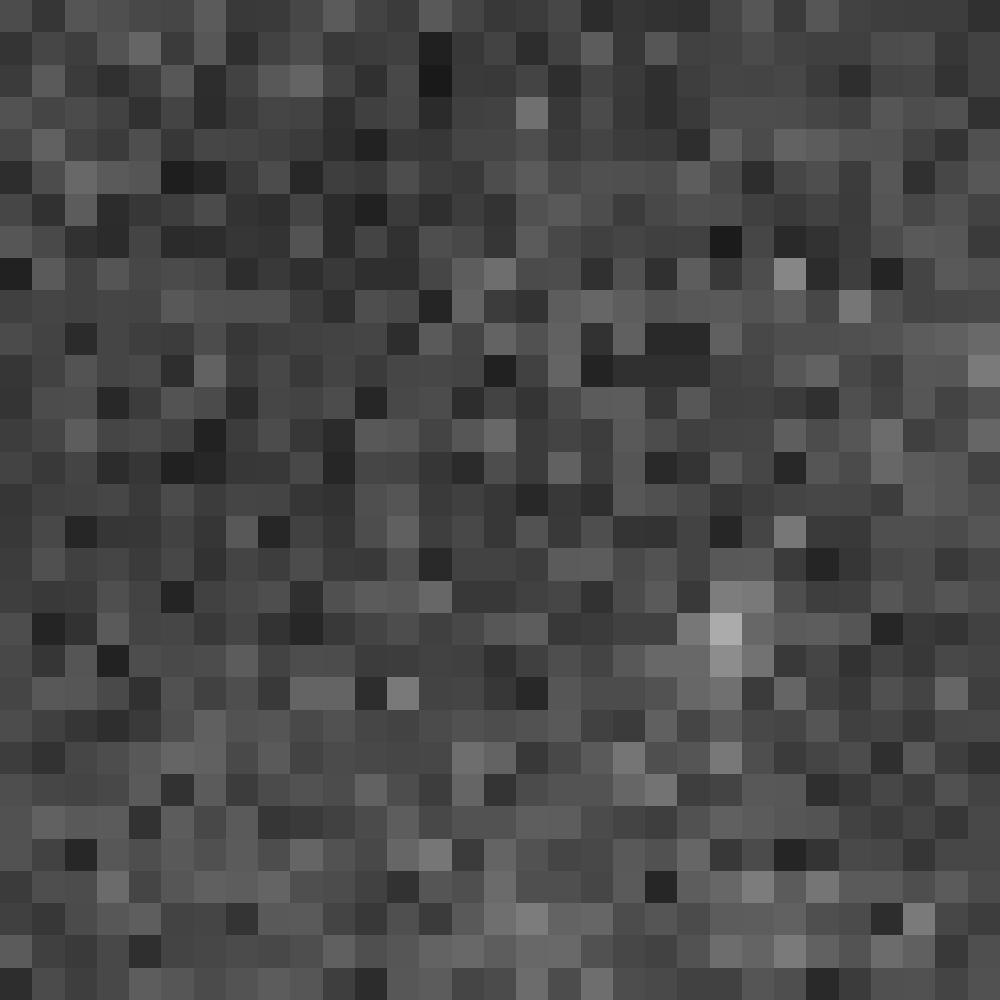

Supplement: Supplementary file 1 [file polymers-11-00084-s001.zip › polymer_kubota_mitsui_JPN_20180107SI/Video S6 lambda DNA clog.gif]
